# Gut Mucosal and Fecal Microbiota Profiling Combined to Intestinal Immune System in Neonates Affected by Intestinal Ischemic Injuries

**DOI:** 10.3389/fcimb.2020.00059

**Published:** 2020-02-25

**Authors:** Lorenza Romani, Federica Del Chierico, Maria Chiriaco, Silvia Foligno, Sofia Reddel, Guglielmo Salvatori, Cristina Cifaldi, Simona Faraci, Andrea Finocchi, Paolo Rossi, Pietro Bagolan, Patrizia D'Argenio, Lorenza Putignani, Fabio Fusaro

**Affiliations:** ^1^Division of Immunology and Infectious Diseases, University-Hospital Pediatric Department (DPUO), Bambino Gesù Children's Hospital, IRCSS, Rome, Italy; ^2^Unit of Human Microbiome, Bambino Gesù Children's Hospital, IRCCS, Rome, Italy; ^3^Neonatal Intensive Care Unit, Department of Medical and Surgical Neonatology, Bambino Gesù Children's Hospital, IRCCS, Rome, Italy; ^4^Digestive Endoscopy and Surgery Unit, Bambino Gesù Children's Hospital, IRCCS, Rome, Italy; ^5^Department of Medical and Surgical Neonatology, Neonatal Surgery Unit, Bambino Gesù Children's Hospital, IRCCS, Rome, Italy; ^6^Unit of Parasitology and Unit of Human Microbiome, Bambino Gesù Children's Hospital, IRCCS, Rome, Italy

**Keywords:** intestinal ischemia, mucosal microbiota, fecal microbiota, intestinal immune system, microbial markers

## Abstract

**Background and Purpose:** Early life microbiota plays a crucial role in human health by acting as a barrier from pathogens' invasion and maintaining the intestinal immune homoeostasis. Altered fecal microbiota (FM) ecology was reported in newborns affected by intestinal ischemia. Our purpose was to describe, in these patients, the FM, the mucosal microbiota (MM) and the mucosal immunity.

**Methods:** Fourteen newborns underwent intestinal resection because of intestinal ischemia. FM and MM were determined through targeted-metagenomics, diversity assignment and Kruskal-Wallis analyses of Operational taxonomic units (OTUs). The mucosal immune cells were analyzed through cytofluorimetry.

**Results and Conclusion:** Based on the severity intestinal injueris we identified two groups: extensive (EII) and focal intestinal ischemia (FII). FM and MM varied in EII and FII groups, showing in the EII group the predominance of Proteobacteria and Enterobacteriaceae and the reduction of Bacteroidetes and Verrucomicrobia for both microbiota. The MM was characterized by a statistically significant reduction of *Bacteroides*, Lachnospiraceae and Ruminococcaceae and by a higher diversity in the EII compared to FII group. FM showed a prevalence of Proteobacteria, while the Shannon index was lower in the EII compared to FII group. An overall increment in B- and T-lymphocytes and Natural killer (NK) T-like cells was found for EII mucosal samples associated to an increment of TNF-α and INF-γ expressing cells, compared to FII group. FM and MM carry specific signatures of intestinal ischemic lesions. Further research may be crucial to address the role of specific taxa in EII, expecially with reference to inflammation grade and ischemia extension.

## Background

Human microbiota is a complex ecosystem consisting of a total of 10^14^ bacteria and characterized by a genomic content (microbiome), which represents more than 100 times the human genome. Since the perinatal life the microbiota commensals play an important role by acting as a barrier against pathogens, stimulating the development of immune system (IS) (Groer et al., 2014; Putignani et al., 2014). This process allows the host and microbiota to coexist in a relationship of mutual benefit and homeostasis (Putignani et al., 2014). Compared to term newborns, the intestinal microbiota of preterm neonates is characterized by fewer bacterial species, lower diversity and increased proportions of potential pathogens. The low stability of gut microbiota in preterm babies increases the risk over time of failure to maintain the balance between IS and gut with negative impact on the intestinal and health system (Palmer et al., 2007; Groer et al., 2014; Del Chierico et al., 2015; Amenyogbe et al., 2017; Pammi et al., 2017). The immaturity of innate immune function interrupts the normal ability of epithelial barrier to control invasive non-commensal bacteria resulting in higher risk of infection and pro-inflammatory cascade (Groer et al., 2014). The main ischemic and inflammatory diseases described in newborn are necrotizing enterocolitis (NEC), midgut volvulus, focal intestine perforation (Messaoudi et al., 2015; Chen et al., 2016; Wieck et al., 2016). Several published data suggest that in neonates affected by intestinal inflammatory injuries such as Necrotizing Enterocolitis (NEC), a reduced diversity and an altered gut microbiota composition may lead to a pro-inflammatory condition that may play a key role in their pathogenesis (Torrazza and Neu, 2013; Till et al., 2015; Moschopoulos et al., 2018). Despite specific bacteria have not been identified, recent papers have shown that some microbial signatures (e.g., *Clostridium* spp. and *Klebsiella* spp.) may represent a risk factor for NEC in infants (Sim et al., 2015; Dobbler et al., 2017; Rusconi et al., 2017). As described in NEC, intestinal dysbiosis might promote, in the small intestinal lamina propria, a local rising of pro-inflammatory mediators and a regulatory T cells reduction, resulting in alteration of the immune balance, with increase of intestinal permeability and susceptibility to infections (Weitkamp et al., 2013). Furthermore dysbiosis associated to impair IS is linkedto a high risk of late onset neonatal sepsis in very preterm newborns (Maynard et al., 2012; Sherman et al., 2015). In spite of these considerations, the comprehension of the mutual relationship between the intestinal IS and the microbiome in neonatal intestinal ischemic injuries is still unclear (Maynard et al., 2012; Engstrand Lilja et al., 2015). While there is a wide literature about intestinal microbiota in NEC, there are few data about the microbiota in the other inflammatory and ischemic diseases such as volvulus, and spontaneous intestinal perforation (Wieck et al., 2016). Therefore, we decided to study the mucosal and fecal microbiota composition and the mucosal immunity in newborns affected by ischemic and inflammatory intestinal diseases regardless the type of a specific disease but based on the ischemic intestinal damage with the need for intestinal resection. Our aim was to identify and propose potential microbial and immunological markers of severity in this type of intestinal damage.

## Methods

### Patient Population

Full term and premature newborns affected by intestinal ischemic injuries requiring intestinal surgery, admitted to the Medical and Surgical Neonatal Intensive Care Unit (Bambino Gesù Children's Hospital) between September 2016 to July 2017, were enrolled after obtaining informed consent from parents. Exclusion criteria consisted in previous intestinal surgery. Intestinal Ischemia was defined extensive (EII) in case of pan-enteric injuries (i.e., necrotizing enterocolitis), and focal (FII) in case of involvement of a specific intestinal segment such as jejunum, ileum, ileocecal valve or colon (i.e., isolated ileal perforation). Full thickeness biopsies were obtained from the wall near ischemic zone in case of FII and from intestinal wall with apparent residual vascularization near to colliquative areas in case of EII.

Nine fecal samples from healthy full term newborns of the age range from 1 to 120 days were also collected as age-matched controls (CTRLs). Patient information including demographic data, prenatal course, birth history, breast feeding, antibiotic therapy, parental nutrition administration, and indications for surgical intervention were collected from the medical records. The study was approved by the Bambino Gesù Children's Hospital ethics committee (*1196_OPBG_2016*).

### Mucosal and Fecal Microbiota

Mucosal and fecal samples were collected during surgery and immediately stored at −80°C until further processing.

DNA, from 200 mg of stools and from 1 ml of enteral washing samples, was extracted using QIAmp Fast DNA Stool mini kit (Qiagen, Germany), while DNA from intestinal mucosa (5 mm^2^) was extracted by the EZ1 Bio robot using EZ1 tissue kit following the manufacturer's instructions.

The variable region V3–V4 of the 16S rRNA gene (~460 bp) was amplified by using the primer pairs 16S_F 5′-(TCG TCG GCA GCG TCA GAT GTG TAT AAG AGA CAG CCT ACG GGN GGC WGC AG)-3′ and 16S_R 5′-(GTC TCG TGG GCT CGG AGA TGT GTA TAA GAG ACA GGA CTA CHV GGG TAT CTA ATC C)-3′ as described in the MiSeq rRNA Amplicon Sequencing protocol (Illumina, San Diego, CA). The PCR reactions were set up following the manufacturer's protocol and using a 2x KAPA Hifi HotStart ready Mix (KAPA Biosystems Inc., Wilmington, MA, USA). DNA amplicons were cleaned-up by AMPure XP beads (Beckman Coulter Inc., Beverly, MA, USA). A second amplification step was performed to obtain a unique combination of Illumina Nextera adaptor-primers. The final library was cleaned-up using 50 ul of AMPure XP beads, quantified using Quant-iT™ PicoGreen® dsDNA Assay Kit (Thermo Fisher Scientific, Waltham, MA) and diluted in equimolar concentrations (4 nM). Samples were pooled together before the sequencing on an Illumina MiSeqTM platform according to the manufacturer's specifications to generate paired-end reads of 300 base-length.

Raw reads were filtered for their quality, read length and chimera presence by QIIME v1.9 pipeline (Caporaso et al., 2010). Obtained cleaned sequences were clustered into Operational Taxonomic Units (OTUs) with a 97% of clustering threshold of pairwise identity (Caporaso et al., 2010). OTUs' representative sequences were aligned (MSA) using PyNAST v.0.1. program (Edgar, 2010) against Greengenes 13_08 database with a 97% of similarity for bacterial sequences. α- and β- diversity were computed by Phyloseq package R software. All metagenomic raw data have been uploaded in ncbi.nlm.nih.gov database. The project code is PRJNA586856.

### Lamina Propria Mononuclear Cells (LMPC) Isolation From Mucosal Samples

Mucosal samples addressed to LMPC isolation were collected during surgery and immediately stored in saline solution at 4°C until further processing. Mucosal samples were washed in HBSS (Euroclone) supplemented with antibiotic mixture (1% Gent/Pen/Strep) and EDTA (6.7 mM) for 45 min at 37°C using a magnetic stirrer. After centrifugation (1,200 rpm/5 min), supernatants were discarded and pellets washed for 3 × 5 min at 37°C on a magnetic stirrer. The samples were digested with Liberase Tm (2 mg/10 mL; Roche) and DNase I (2 mg/10 mL; Roche) in HBSS with 1% of penicillin/ streptomycin/ gentamicin for 45 min at 37°C and 5% CO_2_ on a magnetic stirrer (low speed). LMPC suspensions were then passed through a 100 μm cell strainer filter (Becton Dickenson) and washed 3 times (5 min/1,200 rpm) prior to plating in RPMI-1640 and 10% FBS.

### Flow Cytometry Studies (Fluorescence-Activated Cell Sorting—FACS)

LMPC were stained for surface expression of immune-cells using the following antibodies from BD and Miltenyi: 7AAD (cell viability), CD19-APC, CD3-PERCP, CD56-PE, CD14-FITC, CD103-APC-CY7, CD14-FITC, CD11c-PE, HLADR-PERCP. Intracellular staining for CD68-APCCY7 was performed using BD Cytofix/Cytoperm™ (554722) according to manufacturer instruction.

LMPC were incubated at 37°C/5% CO_2_ over night and then investigated for the presence of T regulatory cells using Anti-Human Foxp3 Staining Set PE (eBioscence, 72-5776). Briefly, cells were stained with CD4-APC (BD), CD25-PE (BD), and CD127-PECY7 (BD) for 20 min at +4°C, washed twice, fixed/ permeabilized (Working solution 1:4) for 30 min at room temperature (RT) and washed two times with 1 × Permeabilization Buffer (5 min/ 1,200 rpm). Cells were then incubated with anti-Foxp3-PE (1:40) for 30 min at RT and protected from light. After washing, cells were resuspended in an appropriate FACS-buffer and acquired on flow-cytometry.

LMPC were incubated at 37°C/5% CO_2_ over night in presence of Brefeldin A (10 μg/μl; Sigma) in resting condition and then analyzed for the presence of: IL17-PE (BD), Tumor necrosis factor alpha (TNFα)-PE (BD) and Interferon gamma (INFγ) (FITC). Intracellular cytokine staining was performed using BD Cytofix/Cytoperm™ (554722) according to manufacturer instruction.

All samples were acquired with BD FACS Canto II (BD FACSDiva™ software v6.1.3) and results analyzed with FlowJo software (v 8.8.6).

### Statistical Analysis

Data from targeted metagenomics and immunological profiles were compared in EII and FII groups. OTUs, α- and β-diversity and statistical significance (Kruskal-Wallis test) were calculated by QIIME software.

Immunological data, evaluated as the means ± error or whiskers boxes, were analyzed by GraphPad Prism program, version 5 (Graph Pad Software, Jolla) and *t*-test, TwoWay Anova with Bonferroni post-test and Mann Whitney test were used to assess if they were statistically different. A *p* < 0.05 was considered significant.

## Results

From January 2016 to August 2017, 14 patients admitted to Medical and Surgical Neonatal Intensive Care Unit of Bambino Gesù Children's Hospital were enrolled. The patient demographics data are reported in Table 1. Based on the severity of intestinal injuries, patients were divided into two groups: seven patients with extensive intestinal ischemia, EII, (five NEC, one midgut volvulus, one total colonic ischemia) and seven patients with focal intestinal ischemia, FII, (four isolated bowel perforation, three intestinal atresia). We collected seven mucosal samples from both groups and all the 14 samples resulted suitable for the microbiota analysis whereas only four fecal samples for group were adequate for the analysis.

**Table 1:** Clinical characteristics of 14 neonates with intestinal ischemia.

**Newborn**	**Sex**	**Gestational age** **(weeks + days)**	**Birth** **weight (gr)**	**Type of birth^**a**^**	**Age** **(days)**	**Breastfeeding**	**Diagnosis**	**Group^**b**^**	**Type of mucosal sample**	**Histology** **diagnosis**	**Necrotic area**
1.	M	29 + 3	1,250	CS	3	Not	Intestinal perforation	FII	Small bowel (ileum)	Vessels congestion and small foci of hemorrhage in lamina propria	Not reported
2.	M	25	700	CS	5	Not	Intestinal perforation	FII	Small bowel (ileum)	Hemorrhagic enteritis	Not reported
3.	M	35 + 3	1,178	CS	32	Not	NEC	EII	Small bowel (ileum)	Hemorrhagic enteritis	11 cm
4.	M	40 +3	4,270	VB	1	Not	Volvulus	EII	Small bowel (ileum)	Necrotizing enteritis	8 cm
5.	F	34 + 6	2,140	CS	1	Not	Intestinal atresia	FII	Small bowel (ileum)	Atrophic mucosa	Not reported
6.	M	33 + 5	1,800	CS	1	Not	NEC	EII	Left Colon	Necrotizing enteritis	Not reported
7.	F	26	900	VB	72	Not	NEC	EII	Small bowel (ileum)	Necrotizing enteritis	Not reported
8.	F	36 + 5	1,550	CS	2	Not	Intestinal atresia	FII	Small bowel (jejunum)	Vessels congestion of the entire wall	Not reported
9.	M	30 + 1	950	CS	61	Not	Colonic ischemia	EII	Right colon	Necrotizing enteritis	3 cm
10.	F	37 + 6	3,560	CS	120	Not	Intestinal atresia	FII	Small bowel (ileum)	Atrophic mucosa	7 cm
11.	M	26	820	CS	10	Not	Intestinal perforation	FII	Small bowel (ileum)	Vessels congestion and small foci of hemorrhage in submucosa	Not reported
12.	F	25	730	CS	9	Not	Intestinal perforation	FII	Small bowel (ileum)	Vessels congestion and small foci of hemorrhage in lamina propria and submucosa	Not reported
13.	M	29 + 5	1,083	CS	17	Not	NEC	EII	Small bowel (ileum)	Necrotizing enteritis	Not reported
14.	F	39	2,785	CS	7	Not	NEC	EII	Left colon	Necrotizing enteritis	Not reported

a *CS, cesarean section; VB, vaginal birth*.

b *FII, focal intestinal ischemia; EII, Extensive intestinal ischemia*.

### Gut Mucosal Microbiota (MM)

Alpha-diversity analysis not revealed a statistical difference in microbiota richness in MM between EII and FII, however the Shannon index mean value was 3.8 ± 1.6 for EII and 2.2 ± 1.4 for FII. Beta-diversity analysis, calculated by unweighted Unifrac metric, revealed a clear separation between samples from the EII and FII groups (permanova *p* = 0.029) (Supplementary Figure 1A).

**Figure 1:**
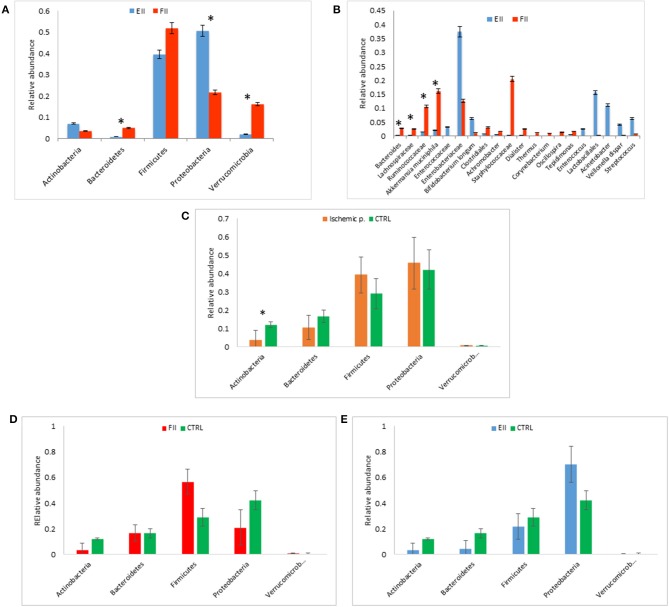
Mucosal microbiota grouped for EII and FII **(A,B)**; FM composition at phylum level for ischemic patients (EII+FII) vs. CTRL group **(C)**, FII vs. CTRL groups **(D)** and EII vs. CTRL groups **(E)**. *p < 0.05.

At phylum level, the comparison between the MM of EII vs. FII showed an increment in Proteobacteria (*p* = 0.049) and Actinobacteria (*p* = 0.42), while Firmicutes (*p* = 0.79), Bacteroidetes (*p* = 0.007) and Verrucomicrobia (*p* = 0.01) were decreased (Figure 1A).

Particularly, at the taxonomic level L6, the EII bacterial distributions revealed the predominance of Enterobacteriaceae followed by Enterococcaceae, Lactobacillales, *Acinetobacter, Streptococcus, Veillonella dispar* and *Bifidobacterium longum* (*p* > 0.05). The FII was characterized by higher levels of *Bacteroides*, Lachnospiracee, Ruminococcaceae*, Akkermansia muciniphila* (*p* < 0.05) and Staphylococcaceae (*p* > 0.05) compared to EII (Figure 1B).

### Fecal Microbiota (FM)

Alpha-diversity indexes of FM showed a higher Shannon index in ischemic patients (mean value 4.1 ± 1.5) compared to CTRL (mean value 2.7 ± 1.3) (*p* > 0.05). Unlike the mucosal samples, alpha-diversity index of FM showed a lower Shannon index in EII (mean value 2.7 ± 0.5), compared to FII (mean value 5.22 ± 0.5) (*p* = 0.01). Beta-diversity analysis, calculated by unweighted Unifrac metric, revealed a clear separation between samples of ischemic patients compared to CTRL group (permanova *p* = 0.001) amongst EII, FII and CTRL groups (permanova *p* = 0.001) and between the EII and FII groups (permanova *p* = 0.032) (Supplementary Figures 1B–D).

The FM of ischemic patients resulted composed by higher content of Proteobacteria and Firmicutes respect the FM of CTRL, while CTRL group showed higher content of Bacteroidetes and Actinobacteria, the latter comparison resulted statistically significant (Figure 1C).

Comparing FM of FII and CTRL, we reported an higher relative abundance of Firmicutes an a lower content of Proteobacteria of FII respect CTRL groups (*p* > 0.05) (Figure 1D). Moreover, the comparison of EII vs. CTRL, showed an opposite trend of these two phyla, in particular Firmicutes were lower in EII and Proteobacteria were higher in EII respect CTRL group (*p* > 0.05) (Figure 1E).

The FM of EII resulted consisting for the majority of Proteobacteria (72%), Firmicutes (20%), while that of the FII was composed mainly by Firmicutes (57%), Proteobacteria (21%), Bacteroidetes (17%) (Supplementary Figure 2A). The comparison of the relative abundances of Proteobacteria and Firmicutes resulted statistically significant (*p* < 0.05). Taxa distribution in ischemic patients was characterized by a lower relative abundance of Enterobacteriaceae respect the CTRL group (Supplementary Figure 3A), also reported for FII vs. CTRL comparison (Supplementary Figure 3B). On the contrary in EII vs. CTRL comparison the Enterobateriaceae resulted higher in EII respect CTRL (Supplementary Figure 3C).

The FM bacterial distributions in the EII group revealed a similar profile to the MM composition with the predominance of Enterobacteriaceae. The FII group was characterized by higher levels of Ruminococcaceae, *Bacteroides*, Lachnospiracee and Staphylococcaceae than EII group (Supplementary Figure 2B). Taxa distributions for each patient were reported in Supplementary Figure 4.

### Immunological Profile of Mucosal Samples

**Figure 2:**
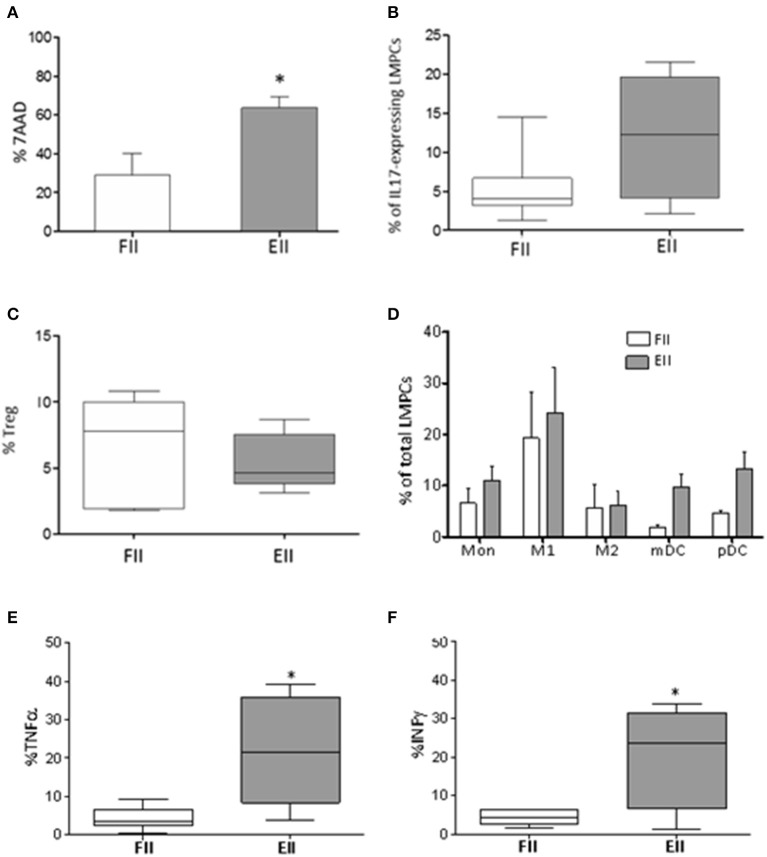
Immunological profile of mucosal samples. **(A)** 7AAD staining of necrotic LMPCs. Results were represented as the mean percentage ± standard error of the mean calculated of total LMPCs, *P < 0.05 Unpaired-two tailed *t*-test; **(B)** percentage of IL17-expressing LMPCs evaluated *ex-vivo* by intra-cellular FACS study; **(C)** percentage of Treg evaluated as CD25+CD127lowFoxp3+ within CD4 T cells; extremities of the whiskers boxes **(B,C)** represent minimum and maximum values; the bottom and top of the box represent the first and the third quartiles, respectively; the horizontal bar in the box represents the median value. **(D)** distribution of myeloid cells in patients; **(E,F)** levels of TNF-α and INF-γ evaluated in resting LMPCs. *P < 0.05 Unpaired-two tailed *t*-test. Monocyte, (Mon) CD14; M1 macrophages, HLADR+CD68+; M2 macrophages, HLADR-CD68+; mDC, CD11c+HLADR+; pDC, CD11c-HLADR+. Extremities of the whiskers boxes **(E,F)** represent minimum and maximum values. The bottom and top of the box represent the first and the third quartiles, respectively. The horizontal bar in the box represents the median value.

LMPC from gut mucosal samples were initially evaluated for cell viability by 7-aminoactinomycin D (7-AAD). According to inflammatory condition, EII group showed an overall increment in B- and T-lymphocytes and Natural killer (NK) T-like cells (Table 2), and increased necrotic cells (28.9 ± 11.4 vs. 63.68 ± 5.8) (Figure 2A) compare to FII group. Interestingly, CD3+ T cell of non-mucosal origin (CD103-) were significantly increased in the EII group (35% ± 9 vs. 15.5% ±4.9; *p* < 0.05). Also an increment of IL17-expressing cells (12% ±4 vs. 5.3% ± 1.8) and a decrease of Tregs (5.4% ± 0.9 vs. 6.6% ± 1.5) were observed for the EII group compared to FII group (Figures 2B,C).

**Table 2:** Percentage mean value and Standard Error Mean (SEM) of immune-cells from patients' LMPC.

	**FII**		**EII**		***P value***
	***Mean***	***SEM***	***Mean***	***SEM***	
B cells (CD 19+)	3.0	1.3	17.6	9.2	NS
T cells (CD3+)	15.5	4.9	35.0	9.0	*p* < 0.05
NK cells (CD56+)	6.8	2.7	9.7	2.1	NS
NKT cells (CD3+CD56+)	2.6	0.8	8.4	2.0	NS
Mucosal Tcells (CD3+CD103+) gated CD3+	7.6	4.9	8.8	3.9	NS

NS, not statistically significant. *p < 0.05, Two-Way Anova with Bonferroni post-test.

Because several innate cells modulate intestinal homeostasis by enhancing or suppressing T cell immune response (Okumura and Takeda, 2016), we studied the myeloid compartment. As showed in Figure 2D both groups of patients had monocytes in the gut. Interestingly both myeloid and plasmacytoid DC (m-DC and p-DC) were increased in EII (mDC 9.7% ± 2.6 and pDC 13.3% ± 3.1) compared to FII group (mDC 1.9% ± 0.5 and pDC 4.6% ± 0.5), although not significant.

Finally, according to inflammatory condition, EII group showed an increment of TNF-α (21.9 ± 6.3% vs. 4.3 ± 1.2% *p* < 0.015^*^) and INF-γ (20.5% ± 6.9 vs. 4.3% ± 0.7 *p* < 0.019^*^) expressing cells (Figures 2E,F), compared to the FII group.

## Discussion

Nowadays it is well-known the essential role of gut microbiota and its interaction with the mucosal and systemic IS in human health. In neonates, the intestinal colonization plays a key role in the development and fine-tuning of the intestinal immune responses. However, the mechanisms of the acquisition of this microbial community during infancy are poorly understood (Renz et al., 2011; Fulde and Hornef, 2014; Lapthorne et al., 2015). In preterm newborns, there is a fragile relationship between the immature epithelial barrier, the gut microbiota and the gut immune system with high risk of dysbiosis, with consequent exposition to potential pathogens and development of severe inflammatory responses and intestinal injuries, such as NEC (Renz et al., 2011; Pammi et al., 2017). Several studies have been conducted trying to describe the microbiota profile potentially involved in the development of intestinal ischemic injuries. In our opinion, a limitation of these investigations is represented by analysis of only stool samples with consequent profiling of the non-adherent bacteria into the gut. The FM, otherwise than the MM, represents an average of the luminal contents with potential loss of intestinal specific-site microbial or immunologic information seven).

In this study, to our knowledge for the first time, we analyzed and compared the microbiota and immune profile of the mucosal samples from specific intestinal sites performed in neonates affected by intestinal ischemic injuries, trying to identify a specific intestinal microbiota and immunologic signature that might be involved as trigger of extensive intestinal damage. Several differences in the gut microbiome profile between mucosal and fecal samples from the EII and FII groups were observed. In patients with EII the MM and FM were characterized by reduction of microbial diversity, with increase in Enterobacteriacae and reduction in *A. muciniphila*, according to the recent literature that describes a correlation between NEC and Enterobacteriaceae, especially *Klebsiella pneumoniae, Citrobacter kruseri*, and *Escherichia coli* (Morrow et al., 2013). From this first observation, we might speculate that, the reduction of microbial diversity in MM and FM, associated to the presence of some microbes in the preterm newborn, such as Enterobacteriaceae, might represent a potential risk factor for the development of inflammatory bowel injuries.

Regarding *A. muciniphila*, this microorganism is an intestinal bacterium isolated a decade ago from a human fecal sample. Interestingly, reduced levels of *A. muciniphila* have been observed in patients with inflammatory bowel diseases (IBD) and metabolic disorders, suggesting that it may have potential anti-inflammatory properties. Furthermore, *A. muciniphila* communicates actively with intestinal IS stimulating the proliferation of anti-inflammatory regulatory T cells in mice. To date, no data have been reported yet about the correlation between A. *muciniphila* and NEC (Derrien et al., 2017). Based on our findings we can suppose that the reduction or absence of *A. muciniphila* on the intestinal mucosa might act as a marker of severe intestinal injury.

Recently, among the Firmicutes, a predominance of V. *dispar* has been described in patients affected by IBD and primary sclerosing cholangitis (Altomare et al., 2018). In our study a preponderanceof *V. dispar* was found in the MM and FM of patients with EII. This result may suggest the utility of further investigation to confirm our data and identify these bacteria as marker of intestinal injury.

The study of immune phenotype performed in both groups of patients revealed that all subsets of immune cells including B, NK and dendritic cells were increased in neonates with EII compared to FII ones, although not significant. In particular, we reported a significant increase of non-mucosal CD3+CD103- T cell as consequence of influx of the systemic activated T cells to inflamed mucosa. Furthermore, the monocytes found in the gut support the hypothesis that they moved toward the intestine in response to tissue damage and then rapidly differentiate into pro-inflammatory M1- and M2-type macrophages. According to data previously reported (Denning et al., 2017) we found in EII patients a rise in IL-17 expressing cells with a slight decrease in T-reg cells. These data support the importance to maintain a correct balance between Th17 and T-reg in order to control/regulate the local inflammatory response. As awaited, cells expressing pro-inflammatory cytokines such as TNF-α and INF-γ, were significantly increased in neonates with EII (De Plaen, 2013). The role of TNFα in the pathogenesis of NEC is well-documented (Baregamian et al., 2009), however that of INF-γ is not clear. Although to date there are no evidences about the role of INF-γ in initiating the inflammatory response in the gut, to regulate the host defense against pathogens, it is well-known their activity in several signaling pathways, including leukocyte migration, epithelial permeability and cell death (Beaurepaire et al., 2009). Moreover several studies performed on animal models demonstrate a link between high level of INF-γ and the development of NEC (Nadler et al., 2000). In particular, the INF-γ know-out mice models are protected against NEC, showing increased epithelial cell restitution compared to wild-type controls when exposed to NEC condition (Leaphart et al., 2007).

Interestingly, in our study a higher production of TNF-α and INF-γ were found in neonates with EII in which the Enterobacteriacae were predominant. Considering these results, we hypothesize the association between the Enterobacteriaceae and a proinflammatory immune response in neonates affected by an intestinal damage. We might speculate that some gut microbiota specific bacteria, such as Enterobacteriaceae, could promote a proinflammatory response. This event could be crucial in determining and maintaining the inflammation and promoting the ischemia extension. This evidence actually is supported by the recent paper on Enterobacteriacae distribution and IgA –bound bacteria in neonates affected by NEC in which fecal samples were associated with less IgA-bound bacteria and increasing domination by Enterobacteriaceae (Gopalakrishna et al., 2019). The main limitations of our study are the sample size and the wide heterogeneity of the study group. Increasing the number of neonates in the study and investigating differences in microbial functionality associated to immunological function will provide further insight in understanding the disease pathogenesis and severity, and the role of the risk factors for the development of the ischemic intestinal injuries. Linking the variations of the microbiota diversity, structure and ecology to disease severity will help to identify new means of using the gut microbiome role.

## Conclusion

The study highlights the relevance of specific gut microbiota signatures in neonatal bowel ischemic events. A microbiota profile rich in Enterobacteriaceae may be related to a proinflammatory response by the immature IS resulting in homeostasis disruption. A FM profile characterized by a reduction of *A. muciniphila* and an abundance of *V. dispar*, might be investigated as a target of intestinal injuries in neonates. Exploring differences in gut microbial functionality and IS response will provide further evidences in order to transform microbiota signatures into microbial ischemia markers.

## Data Availability Statement

This article contains previously unpublished data. All metagenomic raw data contained in can be found in NCBI, accession number PRJNA586856.

## Ethics Statement

The studies involving human participants were reviewed and approved by Bambino Gesù Children's Hospital Ethics Committee. Written informed consent to participate in this study was provided by the participants' legal guardian/next of kin.

## Author Contributions

FF, PD'A, GS, AF, and LR contributed to the conception and design of the research. MC, FD, SR, CC, LR, SFo, and FF contributed to acquisition, analysis and interpretation of the data. LR, SFo, MC, and FD drafted the manuscript. FF, AF, MC, and FD critically revised the manuscript. PB, PR, FF, LP, LR, PD'A, and SFa agreed to be fully accountable for ensuring the integrity and accuracy of the work. All authors read and approved the final manuscript.

### Conflict of Interest

The authors declare that the research was conducted in the absence of any commercial or financial relationships that could be construed as a potential conflict of interest.

## Abbreviations

NEC: Necrotizing Enterocolitis
OTUs: Operational taxonomic units
LMPC: Lamina propria mononuclear cells
TNFα: Tumor necrosis factor alpha
INFγ: Interferon gamma.
