# Supplementary material for: Gut Mucosal and Fecal Microbiota Profiling Combined to Intestinal Immune System in Neonates Affected by Intestinal Ischemic Injuries
Source: Front Cell Infect Microbiol. 2020 Feb 25;10:59. doi: 10.3389/fcimb.2020.00059 (PMC7052114; doi:10.3389/fcimb.2020.00059)

**Supplementary Material**

**Supplementary Figure 1**: Panel A. Beta diversity of MM in EII and FII. Panel B. Beta diversity of FM in ischemic patients and CTRL. Panel C. Beta diversity of FM in EII and FII. Panel D. Beta diversity of FM in EII, FII and CTRL.


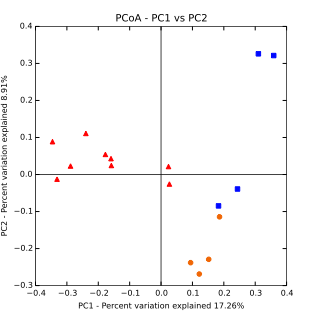


FM CTRL

FM FII

FM EII


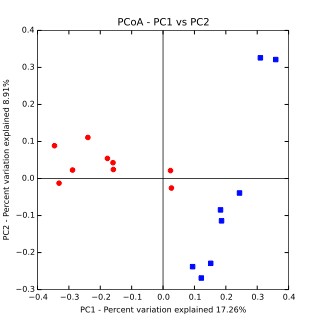


FM CTRL

FM Ischemic patients

A

B

MM EII

MM FII

FM EII

FM FII

C

D

**Supplementary Figure 2:** FM composition at phylum level (**Panel A**) and at species level (**Panel B**) for EII versus FII. (*) p value <0.05.


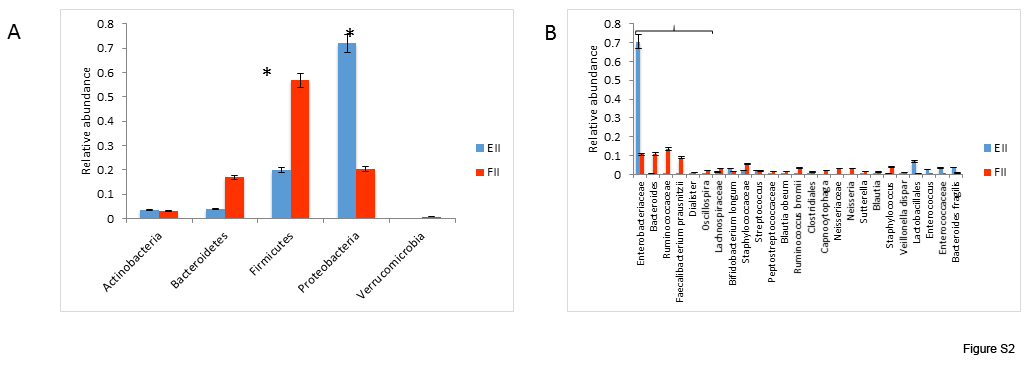


**Supplementary Figure 3**: FM taxa distribution for ischemic patients versus CTRL group (**Panel A**), FII versus CTRL groups (**Panel B**) and EII versus CTRL groups (**Panel C**). (*) p value <0.05.


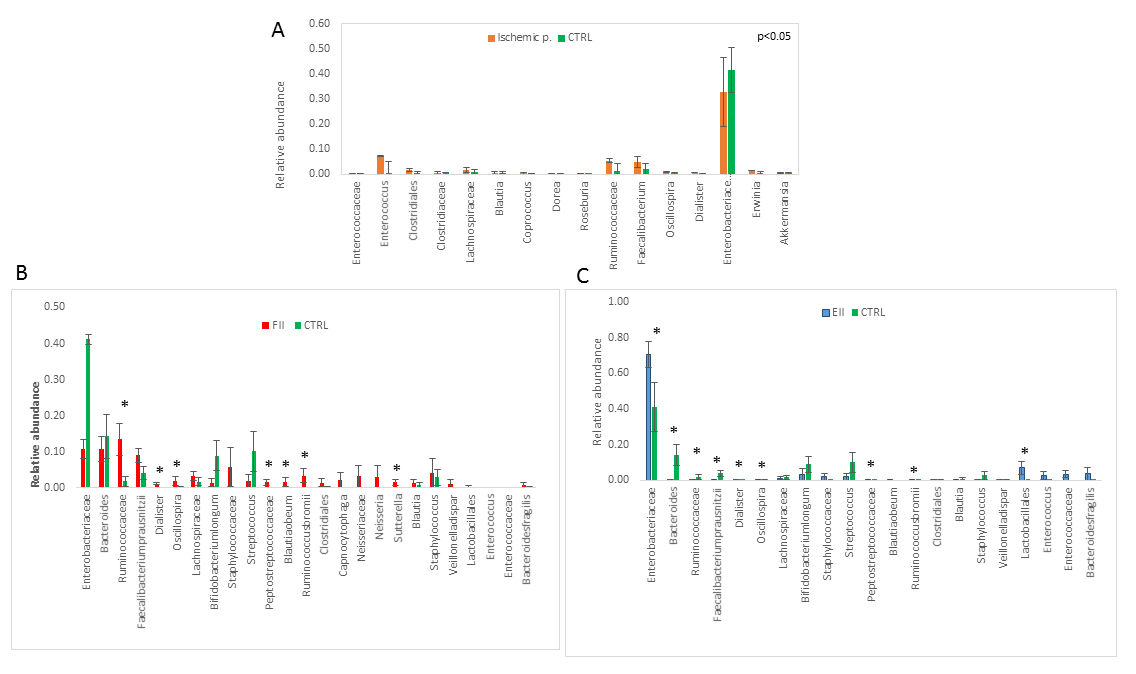


**Supplementary Figure 4**: FM taxa distribution of patients and CTRL subjects.


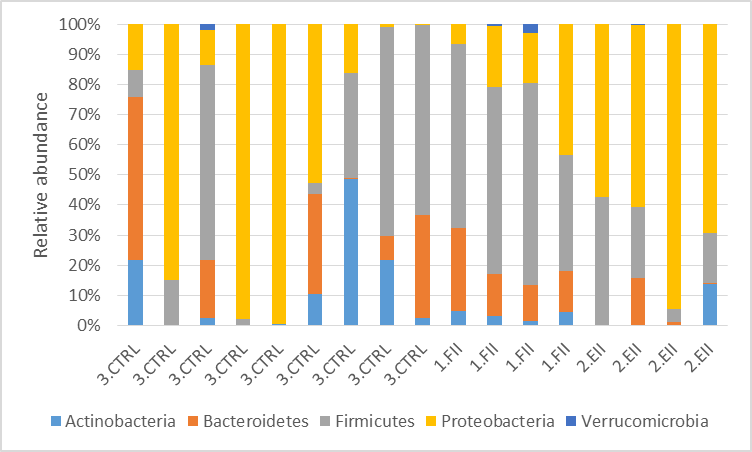

Supplement: Supplementary file 1 [file Data_Sheet_1.docx]
